# Determination of Volatile Compounds in Nut-Based Milk Alternative Beverages by HS-SPME Prior to GC-MS Analysis

**DOI:** 10.3390/molecules24173091

**Published:** 2019-08-26

**Authors:** Natalia Manousi, George A. Zachariadis

**Affiliations:** Laboratory of Analytical Chemistry, Department of Chemistry, Aristotle University of Thessaloniki, 54124 Thessaloniki, Greece

**Keywords:** HS-SPME, milk alternatives, nut-based drinks, almond milk, peanut milk, walnut milk, volatile compounds, gas chromatography, GC-MS

## Abstract

A reliable Headspace-Solid Phase Microextraction (HS-SPME) method was developed for the determination of polar volatile components of commercial nut-based milk alternative drinks prior to Gas Chromatography–Mass Spectrometry (GC-MS) analysis. Under the optimum extraction conditions, a divinylbenzene (DVB)/Carboxen™ CAR)/polydimethylsiloxane (PDMS) fiber was used and 2 mL of sample was heated at 60 °C for 40 min under stirring, without salt addition. Ten compounds from different chemical classes (heptane, a-pinene, toluene, 2-methylpyrazine, 3-heptanone, heptanal, 2-octanone, 1-heptanol, benzaldehyde and 1-octanol) were chosen as model analytes for quantification. Limits of detection and limits of quantification were found to be 0.33–1.67 ng g^−1^ and 1–5 ng g^−1^, accordingly. Good linearity, precision and accuracy were obtained as well as a wide linear range. The proposed method was successfully applied to various beverages including almond milk, walnut milk, peanut milk and almond chocolate milk. More than 70 volatile compounds were detected in the different samples. Most of the detected volatiles were aldehydes, ketones and alcohols. This technique can be used for the determination of volatile compounds in nut-based beverages, to detect compositional changes during storage and technological treatment used for their production.

## 1. Introduction

Plant-based or non-dairy milk alternatives is a fast-growing food product category of functional and specialty beverages that are, nowadays, widely consumed across the world. The origin of the increasing popularity of these products includes cow milk allergies, lactose intolerance, calorie concern, prevalence of hypercholesterolemia as well as increasing preference for functional beverages and vegan diets. [1] Therefore, various alternatives to cow milk products have been developed. Soy and oat-based drinks were, until recently, the most popular milk alternatives. In recent years, several nut-based beverages such as peanut, almond, hazelnut, tiger nut or walnut beverages have become commercially available. Health benefits of such products include the high content of dietary fibers, phytochemicals and vitamins in combination with their relatively low caloric content. Therefore, such products are widely consumed nowadays [1,2,3,4]. 

The flavor of these drinks can be mainly attributed both to the nuts used for their production and to the production process that was implemented [5]. Flavor is the combination of taste and olfaction, and it is a very critical factor that can influence the consumer’s acceptance of foods [6,7]. The profile of volatile compounds is directly associated with food flavor. Ketones, aldehydes, alcohols, alkanes and terpenes are the most common chemical classes of volatiles that can be found in nuts. Moreover, pyrazines, furans and furfurals can be generated during heat treatment processes such as nut roasting [5]. Therefore, it is important to know the typical chromatographic pattern of various food products to identify changes during processing and storage and to relate the food flavor with its volatile composition [6,7].

Solid-phase microextraction (SPME) is a well-established technique for the determination of volatile compounds in nuts [7,8,9] and nut products [5,10,11]. This technique was developed by Pawliszyn and co-workers in 1990 [12,13]. With this sample preparation technique, the analytes are directly extracted and preconcentrated to the outer coating of a fused-silica fiber [14]. As a result, reduction of matrix interference and analyte preconcentration can take place in only one solvent-free stage followed by thermal desorption of the analytes into the Gas Chromatograph injection port [15].

The headspace-SPME (HS-SPME) technique has been the most popular approach for volatile compounds profiling in various food matrices over previous years [6,16,17,18,19,20]. The critical factors that affect the HS-SPME technique depend on analyte properties as well as on sample matrix and should be optimized. These factors are the fiber coating, the extraction temperature, the stirring speed, the equilibration and extraction time and the ionic strength of the solution [6,21].

A few research articles regarding the determination of volatile compounds of nut-based beverages are reported in the literature [5,11]. However, these articles are focused on beverages derived by certain nuts (i.e., almond and tiger nut) and they were not applied to a wide variety of nut-derived beverages. Moreover, the quantification of volatiles of these methods are based on only one chemical compound. Thus, the aim of this research was the development, optimization and validation of a reliable analytical HS-SPME-GC-MS method for the determination of volatile components of a wide range of nut-based beverages such as almond, peanut and walnut beverages. Representative chemical compounds of most classes of volatiles were used for the first time for volatiles’ quantification in nut-based milk’s alternative beverages. To the best of our knowledge, the volatile profile of peanut and walnut milk alternatives beverages is examined for the first time. 

## 2. Results and Discussion

### 2.1. Optimization of HS-SPME Procedure

The method optimization was performed by following the well-established one-factor-at-a-time approach. The effects of extraction time (10–60 min), extraction temperature (25–70 °C), salt addition (0–1.5 g of NaCl), sample volume (1–4 mL) and sample stirring (500–1000 rpm) were investigated. The signals of an alkane (heptane), an alcohol (1-Octen-3-ol), a ketone (4-Methyl-3-penten-2-one) and two aldehydes (hexanal and benzaldehyde) were monitored for the optimization. These analytes were selected due to their high abundance in the volatile compounds in nut-based milk’s alternative beverages. 

#### 2.1.1. Effect of Extraction Time

**Figure 1:**
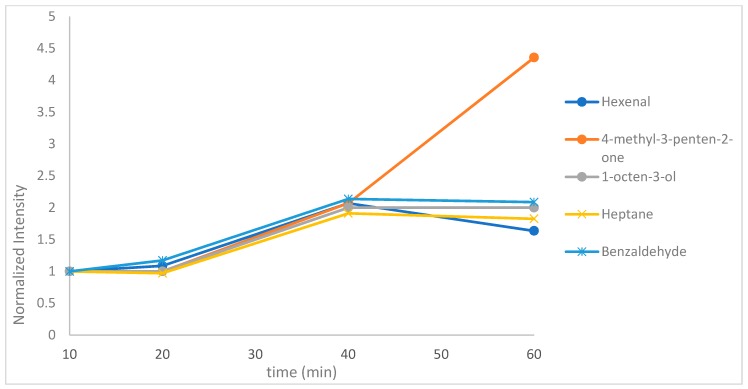
Effect of extraction time on hexanal, 4-methyl-3-penten-2-one, heptane, benzaldehyde and 1-octen-3-ol.

Extraction time is a critical factor for equilibrium techniques such as SPME. The HS-SPME extraction time was examined at 10, 20, 40, and 60 min. SPME is an equilibrium technique, therefore the maximum amount of analyte that can be extracted is reached at equilibrium time. Figure 1 shows the intensity of heptane, benzaldehyde and hexenal/4-methyl-3-penten-2-one/1-octen-3-ol at various extraction times. As it can be observed, at 40 min several analytes can be detected and the signal for most of them is higher. Only for the ketone does the intensity continues to increase with the increasing of time. This can be attributed to lower diffusion coefficient which leads to slower mass transfer and longer equilibrium time compared to the rest analytes [22]. However, for heptane and benzaldehyde, the intensity is slightly reduced with the increasing of time to more than 40 min. As a compromise, 40 min was finally chosen as extraction time. 

#### 2.1.2. Effect of Extraction Temperature

**Figure 2:**
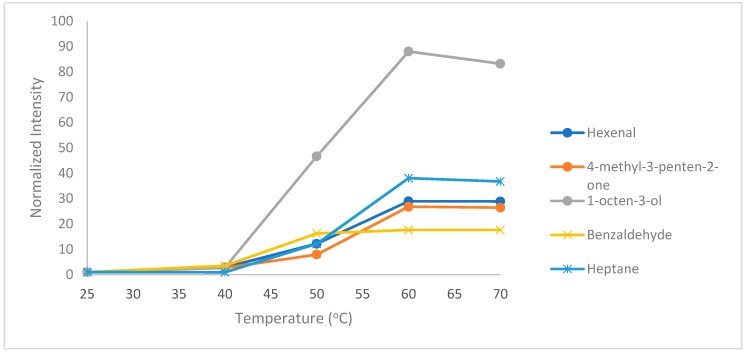
Effect of extraction temperature on hexanal, 4-methyl-3-penten-2-one, heptane, benzaldehyde and 1-octen-3-ol.

The HS-SPME extraction procedure was performed without heating the sample as well as with heating at 25, 40, 50, 60 and 70 °C. For almost all analytes, extraction temperature of 60 °C resulted in higher signal intensity. A further increase in temperature above 60 °C resulted in signal intensity reduction. High extraction temperature is known to release more volatiles in the headspace and assist in the extraction procedure. However, adverse effects, such as decrease of partition coefficients, which prevent the extraction, can also take place [23]. Therefore, 60 °C was selected as the optimum HS-SPME extraction temperature. Figure 2 shows the effect of extraction temperature on the intensity of selected analytes.

#### 2.1.3. Effect of Salt Addition

**Figure 3:**
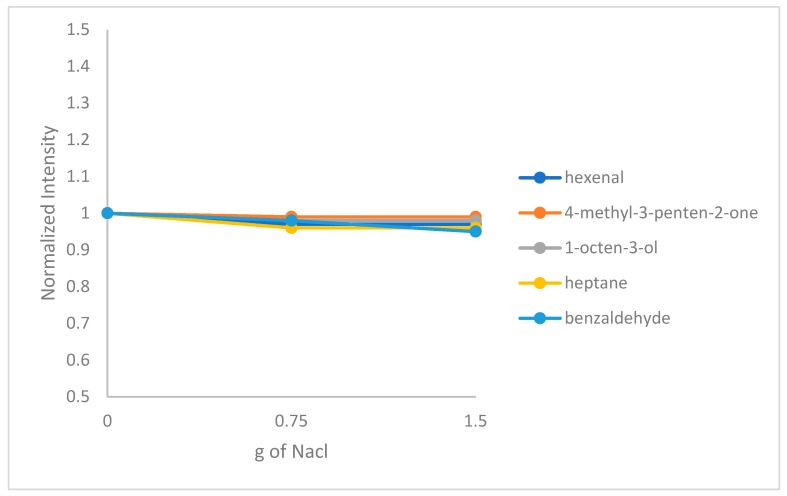
Effect of salt addition on hexanal, 4-methyl-3-penten-2-one, heptane, benzaldehyde and 1-octen-3-ol.

Addition of sodium chloride was tested to check its effect on the HS-SPME procedure. The salting-out method is known to increase the distribution constant between the fiber and the analytes since it minimizes the interaction of the analytes with water and helps the analytes to go to the headspace [22]. An addition of 0.75 g and 1.5 g of sodium chloride, as well as absence of salt, were tested. Figure 3 shows the effect of salt addition in the extraction procedure. It was found that there was no significant effect of sodium chloride addition. As a result, no salt addition was required. 

#### 2.1.4. Effect of Sample Stirring

**Figure 4:**
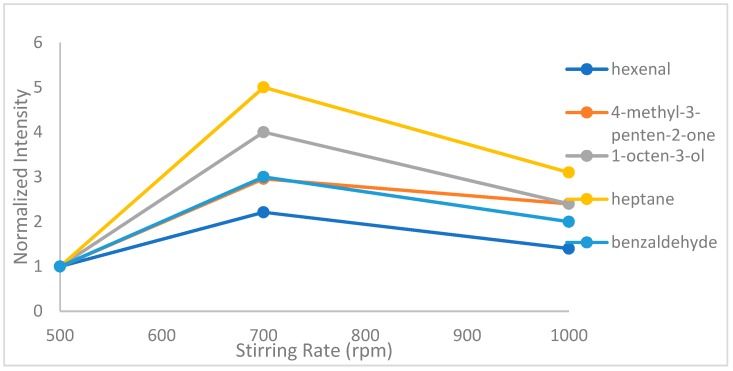
Effect of stirring rate on hexanal, 4-methyl-3-penten-2-one, heptane, benzaldehyde and 1-octen-3-ol.

The detection of several volatile components of nut-based beverages was enabled by stirring the sample by a magnetic stirrer. Figure 4 shows the effect of stirring rate in the extraction procedure. Stirring leads to more effective and faster adsorption of analytes into the fiber which can be achieved due to the assisted volatilization of analytes. Therefore, stirring rate of 500–1000 rpm was tested and a stirring rate of 700 rpm was chosen to obtain higher signals [22].

#### 2.1.5. Effect of Sample Volume 

**Figure 5:**
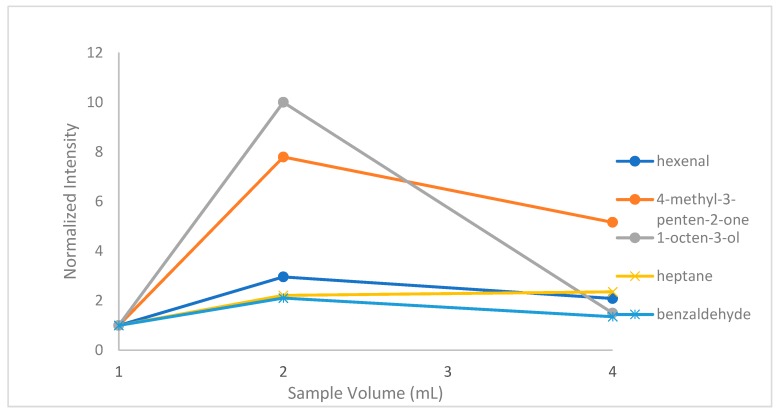
Effect of sample volume on hexanal, 4-methyl-3-penten-2-one, heptane, benzaldehyde and 1-octen-3-ol.

For the HS-SPME extraction procedure, sample volumes of 1, 2 and 4 mL were tested, using 15-mL vials. Figure 5 shows the effect of sample volume in the extraction procedure. The number of adsorbed analytes is generally proportional to sample volume. Therefore, higher sample volume lead to higher analyte extraction. However, fiber overload can have negative effects due to reverse diffusion of analytes from the fiber to the sample. In this experiment, an increase in sample volume from 1 to 2 mL lead to analyte extraction enhancement. However, a further increase from 2 to 4 mL had negative effect in the extraction procedure resulting in optimum sample volume of 2 mL for the examined analytes [24].

### 2.2. Validation

#### 2.2.1. Selectivity

The satisfactory resolution between the chromatographic peaks of target analytes as well as the absence of interferences from the sample matrix indicate that a good selectivity was achieved with the proposed HS-SPME method.

#### 2.2.2. Linearity, Limits of Detection (LODs) and Quantification (LOQs) 

The calibration curves for heptane, a-pinene, toluene, 2-methylpyrazine, 3-heptanone, heptanal, 2-octanone, 1-heptanol, benzaldehyde and 1-octanol are shown in Table 1. For this purpose, spiked samples of different concentrations (10, 25, 50, 100, 250 500, 1000, 5000, 10000, 25000 ng g^−1^) were analyzed. LOQ values ranged from 1.00–5.00 ng g^−1^, while LOD values ranged from 0.33 to 1.67 ng g^−1^. The upper limit of the calibration curves ranged between 2500 and 25000 ng g^−1^, depending on the analyte. To the best of our knowledge, there are no mentioned LOQ and LOD values in the literature for the HS-SPME-GC-MS methods about nut drinks and nut extracts [5,11,25]. As it can be concluded, the developed HS-SPME method has satisfactory linearity range, as well as low LOD and LOQ values.

#### 2.2.3. Accuracy and Precision

Extraction recovery values were used for the assessment of method accuracy at a concentration level of 1000 μg g^−1^ for heptane, a-pinene, toluene, 2-methyl-pyrazine, 3-heptanone, heptanal, 2-octanone, 1-heptanol, benzaldehyde and 1-octanol in a solution containing also 0.5 μg mg^−1^ butyrophenone. The results were between 81.3% and 118.2%, indicating satisfactory method accuracy. For the determination of method precision, means of relative standard deviation (RSD) was employed. For within-day repeatability (n = 5 days), RSD values ranged between 0.4% and 10.7%, while for between-days repeatability (n = 3 × 4 days) RSD values ranged between 1.3 and 12.3%. It can be concluded that the reported HS-SPME method is precise. Table 2 shows accuracy and precision results for the studied analytes.

### 2.3. Real Samples Analysis

All the nut-based beverages used in this study were obtained from the local market in Thessaloniki, Greece. Different varieties of nut-based beverages (almond drink, chocolate almond drink, peanut drink, walnut drink) were analyzed as well as samples from different companies derived from almond. All samples were stored in the refrigerator (+4 °C). Results are illustrated in Table 3. Identification of analytes was performed with (National Institute of Standards and Technology=Environmental Protection Agency=National Institutes of Health) NIST=EPA=NIH Mass spectral library NIST 05. Similarity indices were >95% for the analytes in Table 3. As it can be observed, there are significant differences in the volatile profile of drinks derived by different nuts (i.e., almond, peanut, walnut) as well as of almond drinks from different producers.

**Figure 6:**
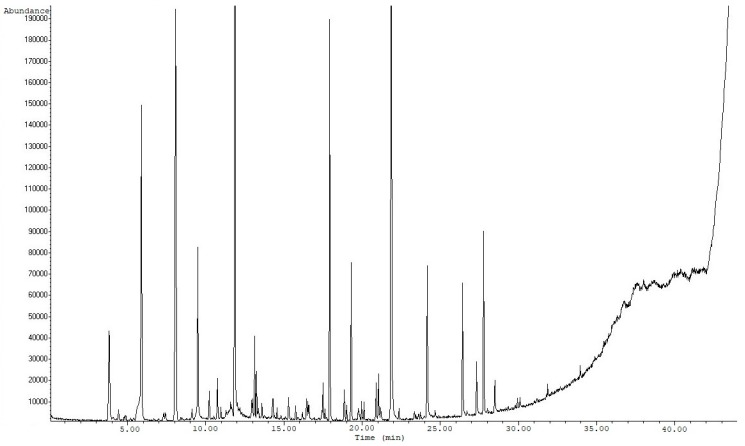
Chromatogram of walnut drink analysis.

As shown in Table 3, the most common volatiles of nut-based beverages belong to the chemical classes of alkanes (such as heptane, 3-methyl-hexane, 2,2-dimethyl-hexane etc.), aldehydes (such as pentanal, hexanal, (E)-2-hexenal, hepanal, octanal, octenal, benzaldehyde etc.), ketones (acetone, 2-butanone, 4-heptanone, 3-heptanone, 2-octanone etc) and alcohols (ethanol, 1-pentanol, 1-hexanol, 1- heptanol, 2-heptanol). Terpenes (such as a-pinene and limonene) were also detected. These compounds have been previously detected in the respective nuts as well as in almond drink [5,8,9]. Pyrazines and furfurals that were detected at the samples can be attributed to Strecker reaction due to the thermal treatment, while furans can be attributed to Maillard reaction [5]. Differences among the samples can be attributed both to the different nuts used for the drinks’ production and to the different parameters of the drinks’ production. Compounds such as styrene were not previously reported in these kinds of products, and therefore migration of the packaging may have occurred [26]. Since commercial samples were analyzed, addition of flavor enhancers may be another reason for the variation of type and concentration of some volatiles. Figure 6 shows a representative chromatogram obtained from walnut drink analysis.

These compounds form the total volatile profile of these products containing not only chemical compounds that form the product’s flavor, but also other volatiles that are present in such drinks, since extraction was performed at 60 °C. 

In order to evaluate the chemical compounds that are responsible for the product’s flavor, extraction was performed at lower temperature. An extraction temperature of 37 °C may better simulate flavor release from the mouth [27]. For Almond Sample 1, analysis was also performed after extracting the volatile compounds at lower temperature (37 °C) instead of the optimum temperature that was finally chosen (60 °C). The compounds that were detected after extraction at this temperature are also shown in Table 3. Twenty-six volatile compounds could be detected at this extraction temperature; most of them were aldehydes and ketones. As a result, by reducing extraction temperature, the volatile compounds of nut derived beverages, that are responsible for the drink’s flavor, can be determined with this method. 

## 3. Materials and Methods 

### 3.1. Chemicals and Materials

All samples of nut-based milks (i.e., almond beverages, walnut beverage, peanut beverage, almond and cacao beverage) were obtained from the local market in Thessaloniki, Greece. All samples were stored in the refrigerator (+4 °C). Butyrophenone (purity ≥99%) was used as internal standard (IS) solution (Sigma-Aldrich, St. Louis, MO, USA). A stock solution of IS was prepared in methanol (Panreac, Barcelona, Spain) at a concentration of 1000 mg L^−1^. A stock standard solution containing heptane (purity >99%, Sigma-Aldrich), a-pinene (purity >95%, Fluka, St. Gallen, Swiss), toluene (purity >99.8%, Sigma-Aldrich), 2-methylpyrazine (purity >99%, Sigma-Aldrich), 3-heptanone (purity >98%, Sigma-Aldrich), heptanal (purity >95%, Sigma-Aldrich), 2-octanone (purity >98%, Sigma-Aldrich), 1-heptanol (purity >98%, Sigma-Aldrich), benzaldehyde (purity >99%, Sigma-Aldrich) and 1-octanol (purity >99%, Sigma-Aldrich) was prepared in methanol (concentration of each analyte 1000 mg L^−1^) and used for the identification of the volatile compounds and method validation. Τhe stock solution was stored in the refrigerator (+4 °C) and it was found stable for up to 2 months. Working standard solutions were prepared daily by diluting appropriate amount of stock standard solution and IS solution in distilled water. 

### 3.2. HS-SPME Procedure

The headspace solid phase microextraction (HS-SPME) was performed with a 23GA Stablefex™ 2 cm-long 50/30 μm DVB/CAR/PDS fiber from Supelco, attached in a manual SPME fiber holder (57330-U, Supelco, Germany). For the HS-SPME procedure, glass vials (15 mL) closed with polytetrafluoroethylene (PTFE) coated silicone rubber septum were used. Prior to analysis, the fibers were preconditioned in the injector port of the GC System according to the manufacturer’s instructions. Under the optimum conditions, an aliquot of 2 mL of the nut-based beverage was transferred into the glass vial and 100 μL of internal standard solution was added to the vial (final IS concentration 0.5 μg g^−1^). The samples were heated at 60 °C and extraction was achieved in 40 min under agitation without any addition of salt. By the time that the incubation temperature was obtained, the fiber was immersed in the vial and one-stage simultaneous extraction and incubation was performed. Every sample was analyzed in triplicate.

### 3.3. Gas Chromatography–Mass Spectrometry (GC-MS) Analysis

An Agilent 6873K Gas Chromatograph coupled with an Agilent 5973 Quadrupole Mass Spectrometric Detector (Hewlett Packard, Waldbronn, Germany) was used for the separation and identification of the analytes. A DB-WAX capillary column (60 m × 0.32 mm, 0.25 μm) column was used as stationary phase, while helium was used as the carrier gas at a flow rate of 1 mL/min. The column temperature program was as follows: 40 °C initial temperature held for 5 min, raised to 100 °C at a rate of 10 °C /min, further raised to 220 °C at a rate of 5 °C /min held for 5 min and finally raised to 250 °C at a rate of 15 °C /min. Total analysis time was 44 min. Analyte desorption took place in the GC injection port at 250 °C for 3 min in splitless mode and the fiber was kept in the injection port for 5 min more for cleaning. Mass analyzer was set on scan mode and the recorded ions were *m/z*:35 to m/z:350. MS source and MS Quad were operated at 250 °C and 130 °C, respectively. Finally, identification of analytes was performed with NIST=EPA=NIH Mass spectral library NIST 05 and the retention time of some volatile compounds was used as reference standards.

### 3.4. Method Validation

The herein developed HS-SPME method was validated in terms of selectivity, linearity, precision, accuracy and sensitivity. Linearity studies were performed by triplicate analysis covering the entire working range. The slope, intercept and coefficient of determination was calculated based on least square linear regression analysis. Limits of detection and quantification were calculated by 3 S/N and 10 S/N ratio. The method accuracy was calculated as the recovery (%) value obtained from the measured concentrations compared to nominal concentrations of spiked solutions using the equation:

$$Recovery\;\left(\%\right)=100\times \frac{Measured\;concentration}{Nominal\;concentration}$$

Within-day repeatability was evaluated by calculating the relative standard deviation (RSD) for five replicate measurements of spiked solutions, while between-days precision and accuracy was assessed by performing triplicate analysis at the same concentration level in four different days. Selectivity of the developed method was assessed by the absence of matrix interference in real samples’ chromatograms and the good resolution between the analytes’ peaks.

## 4. Conclusions

Herein, a HS-SPME method was developed and validated for the determination of polar volatile components of commercial nut-based drinks prior to GC-MS analysis. The proposed method is simple, selective and inexpensive and shows good linearity as well as low LOQ and LOD values. Moreover, the proposed method shows satisfactory accuracy and precision. With the HS-SPME technique, reduction of matrix interference and preconcentration of more than 70 volatile compounds can be achieved. Most of these compounds belong to aldehyde, ketone and alcohol groups. Representative chemical compounds of most classes of volatiles were used for the first time for volatiles’ quantification in nut-based milk’s alternative beverages. The proposed method can be used for the determination of volatile compounds in a wide range of nut-based beverages, to detect changes during storage and technological treatment used for their production and to relate the sensory characteristics of products with their total polar volatile profile.

## Figures and Tables

**Table 1:** Regression equations, linear range, Limits of Detection (LOD) and Limits of Quantification (LOQ) values for the target analytes.

Analyte	Linear Range (ng g^−1^)	Regression Equation	R^2^	LOQs (ng g^−1^)	LODs (ng g^−1^)
heptane	10–5000	y = (0.0311 ± 0.0017)x + (0.0055 ± 0.0047)	0.9942	1.00	0.33
a-pinene	500–10000	y = (0.2883 ± 0.0102)x + 0.507 ± 0.024)	0.995	5.00	1.67
toluene	10–10000	y = (0.7595x ± 0.06167) + (0.1382 ± 0.0141)	0.9983	1.00	0.33
2-methylpyrazine	10–10000	y = (0.3866x ± 0.0360) + (0.0757 ± 0.0068)	0.9963	1.00	0.33
3-heptanone	10–10000	y = (0.3523x ± 0.0152) + (0.0859 ± 0.0761)	0.9944	1.00	0.33
heptanal	100–25000	y = (0.0571x ± 0.0006) + (0.0024 ± 0.0032)	0.9996	1.00	0.33
2-octanone	10–2500	y = (1.0157x ± 0.0508) + (0.0697 ± 0.0623)	0.9925	1.00	0.33
1-heptanol	10–25000	y = (0.8018x ± 0.0104) + (0.0084 ± 0.0003)	0.992	1.00	0.33
benzaldehyde	10–25000	y = (1.644x ± 0.0245) − (0.5051 ± 0.0301)	0.992	1.00	0.33
1-octanol	10–25000	y = (1.979x ± 0.0256) + (0.2554 ± 0.0244)	0.999	1.00	0.33

**Table 2:** Accuracy and precision results of method validation for the determination of volatile compounds in nut-based milk alternative drinks.

Analyte	Within Day (n = 5)			Between Days (3 × 4 days)		
	Found ± SD (μg g^−1^)	RSD (%)	Recovery (%)	Found ± SD (μg g^−1^)	RSD (%)	Recovery (%)
Heptane	966 ± 55	5.7	96.6	984 ± 11	11.0	98.5
a-pinene	1013 ± 108	10.7	101.3	997 ± 12	12.1	99.8
Toluene	1016 ± 80	7.9	101.6	979 ± 78	8.0	97.9
2-methylpyrazine	1157 ± 17	1.4	115.7	1023 ± 18	1.8	102.3
3-heptanone	1123 ± 19	1.7	112.3	1026 ± 34	3.4	102.6
Heptanal	813 ± 24	2.9	81.3	856 ± 33	3.9	85.7
2-octanone	991 ± 12	1.2	99.1	943 ± 12	1.3	94.4
1-heptanol	1182 ± 46	3.9	118.2	1094 ± 81	7.4	109.4
benzaldehyde	1157 ± 52	4.6	115.7	1067 ± 131	12.3	106.7
1-octanol	889 ± 112	1.3	88.9	921 ± 45	4.9	92.1

**Table 3:** Results obtained from commercial drink samples analysis.

Peak No	RT (min)	Compound	Reference Compound	Content (ng g^−1^)				
				Walnut	Peanut	Choco almond	Almond Sample 1	Almond Sample 2
1	4.431	Heptane *	Heptane	112.1 ± 21.2	40.1 ± 6.3	75.4 ± 8.7	79.1 ± 2.1	183.6 ± 9.7
2	4.790	3-methyl-hexane	Heptane	34.1 ±2.3	38.5 ± 1.3	58 ± 9.3	53.4 ± 7.3	47.5 ± 2.7
3	4.886	2,3,3-trimethylpentane *	Heptane	47.2 ± 5.4	40.4 ± 2	88.3 ± 6.2	77.4 ± 5.5	ND
4	5.214	2-methylpropanal	Heptanal	ND	70.5 ± 1.4	12 ± 2.2	11.4 ± 1.1	ND
5	5.617	Acetone	3-heptanone	ND	ND	ND	1.3 ± 0.3	ND
6	5.797	1,2,4-trimethylcyclopentane	Heptane	ND	104.6 ± 11.2	ND	57.6 ± 9.1	ND
7	7.020	2-butanone *	3-heptanone	ND	ND	4.3 ± 0.8	2.1 ± 0.1	ND
8	7.307	2-methyl-butanal *	Heptanal	290.6 ± 11.1	110.6 ± 7.5	2160.2 ± 108.2	1180.6 ± 153.4	220.6 ± 15.3
9	7.424	3-methyl-butanal *	Heptanal	220.4 ± 13.4	60.4 ± 5.4	4920.3 ± 202.3	820.4 ± 56.1	260.3 ± 17.3
10	8.074	Ethanol	1-heptanol	121.2 ± 12.1	1.3 ± 0.2	ND	2.3 ± 0.4	ND
11	8.412	2-ethylfuran	-	>LOQ	ND	ND	ND	ND
12	8.485	2,2-dimethyl-hexane	Heptane	11.1 ± 2.2	ND	ND	1.21 ± 0.2	35.49 ± 3.2
13	9.116	Pentanal	Heptanal	43.3 ± 4.2	>LOQ	230.4 ± 11	2190.6 ± 1.1	770.4 ± 3.3
14	10.219	a-pinene *	a-pinene	11.3 ± 2.1	41 ± 5.4	20.2 ± 6.2	9.9 ± 4.1	9.5 ± 2.2
15	10.739	Toluene *	Toluene	39.3 ± 4.3	24.3 ± 5	19.5 ± 3.1	14.5 ± 2.1	26.1 ± 4.4
16	10.968	2,3-dimethyl-3-hexanone	3-heptanone	Below LOQ	ND	ND	ND	ND
17	11.285	2,3-pentanedione	3-heptanone	1.2 ± 0.1	ND	3.4 ± 0.3	1.7 ± 0.4	1.4 ± 0.2
18	11.59	Dimethyldisulfide	-	>LOQ	ND	>LOQ	>LOQ	>LOQ
19	11.854	Hexanal *	Heptanal	4494.2 ± 48.1	606.2 ± 12.1	254.4 ± 16.3	518.1 ± 23.4	208.5 ± 61.3
20	12.94	4-heptanone *	4-Heptanone	21.1 ± 1.3	32.5 ± 3.6	14.6 ± 4.5	11.1 ± 0.9	10.2 ± 1.3
21	12.99	(E)-3-Penten-2-one *	3-heptanone	ND	14.5 ± 8.9	25.6 ± 7.8	30.4 ± 2.6	ND
22	13.134	4-methyl-3-Penten-2-one *	3-heptanone	124.5 ± 11.6	ND	174.5 ± 13.6	214.5 ± 14.3	ND
23	13.371	3-carene	a-pinene	ND	8.4 ± 0.6	10.2 ± 3.5	ND	ND
24	13.596	3-heptanone *	3-heptanone	25.6 ± 7.3	41.4 ± 9.2	51.4 ± 0.9	1.4 ± 0.1	1.5 ± 0.2
25	14.268	2-butyl-Tetrahydrofuran	-	ND	ND	ND	>LOQ	ND
26	14.299	Heptanal *	Heptanal	8.6 ± 0.9	170.1 ± 9.2	90.2 ± 8.3	9.8 ± 0.1	ND
27	14.556	Limonene	a-pinene	7.1 ± 0.4	6.7 ± 0.2	8.2 ± 0.4	9.7 ± 0.3	11.2 ± 0.5
28	15.083	(E)-2-Hexenal *	Heptanal	ND	ND	ND	80 ± 4	ND
29	15.29	1-ethyl-3-methyl-benzene	Toluene	24.1 ± 2.2	2.1 ± 0.4	1.9 ± 1.2	3.2 ± 0.4	1.5 ± 0.2
30	15.464	1,2,3-trimethylbenzene	Toluene	ND	ND	ND	4.1 ± 0.5	ND
31	15.752	6-methyl-2-heptanone *	3-heptanone	ND	ND	11.9 ± 0.9	19.3 ± 0.5	20.6 ± 1.3
32	15.889	1-pentanol	1-heptanol	11.4 ± 0.6	9.8 ± 0.9	ND	ND	ND
33	16.179	Styrene	a-pinene	9.8 ± 1.2	ND	ND	ND	ND
34	16.227	2-methyl-pyrazine	2-methyl-pyrazine	1.2 ± 0.1	3.4 ± 0.5	ND	2.3 ± 1.2	ND
35	16.541	2-octanone	2-octanone	8.1 ± 0.3	11 ± 1	ND	ND	7.6 ± 0.3
36	16.602	Octenal *	Heptanal	192.6 ± 3.4	181.4 ± 15.7	121.6 ± 13.1	303.4 ± 15.6	181.5 ± 25.1
37	16.666	Octanal	Heptanal	ND	181.1 ± 3.6	ND	ND	ND
38	16.946	1-hepten-3-one	3-heptanone	ND	9.7 ± 0.9	ND	8.7 ± 1.2	5.6 ± 1.1
39	17.205	2-heptanol	1-heptanol	11.4 ± 3.1	12.1 ± 0.4	9.9 ± 1.1	11.1 ± 3.2	12.3 ± 2.1
40	17.428	(Z)-2-heptenal *	Heptanal	ND	ND	22.1 ± 2.5	910.1 ± 43.4	131.3 ± 15.4
41	17.491	2,5-dimethyl-pyrazine	2-methyl-pyrazine	42.1 ± 8.2	10.1 ± 1.6	12.2 ± 3.1	3.3 ± 1.1	ND
42	17.618	2,6-methyl-pyrazine	2-methyl-pyrazine	9.1 ± 0.3	ND	8.5 ± 0.4	7.6 ± 0.3	5.7 ± 0.4
43	17.93	1-hexanol	1-heptanol	554.86 ± 21.6	30.3 ± 6.4	ND	11.2 ± 3.1	ND
44	18.358	Esculetin	a-pinene	ND	ND	ND	7.8 ± 1.1	ND
45	18.737	dimethytrisulfide	-	Below LOQ	>LOQ	>LOQ	>LOQ	>LOQ
46	18.853	Nonanal *	Heptanal	952.1 ± 14.7	121.3 ± 15.4	869.4 ± 13.7	2521.5 ± 76.9	79.1 ± 4.9
47	19.005	2-ethyl-5-methyl-pyrazine	2-methyl-pyrazine	ND	6.9 ± 1.1	12.4 ± 1.8	50.6 ± 4.5	ND
48	19.276	2-nonanone	3-octanone	3.1 ± 0.5	7.8 ± 0.5	10.6 ± 0.9	ND	ND
49	19.845	2,6-diethyl-pyrazine	2-methyl-pyrazine	4.5 ± 0.6	6.6 ± 0.9	7.6 ± 1.1	8.4 ± 0.7	ND
50	19.966	1-octen-3-ol	1-octanol	23.4 ± 2.4	9.8 ± 1.1	12.2 ± 1.5	24.4 ± 3.9	11.6 ± 0.4
51	20.124	1-heptanol	1-heptanol	28.4 ± 5.6	11.6 ± 2.5	52.6 ± 3.2	71.9 ± 7.8	13.6 ± 1.7
52	20.413	Furfural	Heptanal	ND	ND	77.3 ± 9.4	140.1 ± 13.3	ND
53	20.535	2,6-diethyl Pyrazine	2-methyl-pyrazine	ND	ND	3.4 ± 0.7	1.2 ± 0.1	3.8 ± 0.4
54	20.845	2-benzothiazol	Benzaldehyde	1.3 ± 0.4	5.6 ± 1.3	3.2 ± 1.3	4.4 ± 0.9	1.3 ± 0.9
55	20.895	2-ethyl-1-hexanol *	1-heptanol	40.2 ± 3.4	20.2 ± 5.5	10.7 ± 3.1	95.6 ± 2.3	82.3 ± 4.1
56	21.194	5’-amino-5’-deoxy adenosine *	-	>LOQ	>LOQ	>LOQ	>LOQ	>LOQ
	21.858	Benzaldehyde *	Benzaldehyde	1503.5 ± 11.6	119.1 ± 11.5	153.5 ± 4.6	1933.1 ± 22.6	2327.5 ± 36.4
58	21.945	(E)-2-nonenal *	Heptanal	ND	ND	126.5 ± 9.4	176.5 ± 15.1	102.1 ± 13.1
59	22.355	1-octanol *	1-octanol	10.3 ± 0.1	9.4 ± 0.7	10.1 ± 0.4	50.3 ± 5.1	ND
60	23.353	6-methyl-(E)-3,5-heptadien-2-one	1-heptanone	>LOQ	>LOQ	9.6 ± 0.2	11.5 ± 1.3	>LOQ
61	23.635	1-(2-pyridinyl)-ethanone *	2-methyl-pyrazine	ND	3.1 ± 0.4	4.8 ± 0.6	63.9 ± 10.1	Below LOQ
62	24.68	Internal Standard	-	-	-	-	-	-
63	24.758	4-methyl-benzaldehyde	Benzaldehyde	ND	ND	3.4 ± 0.1	ND	ND
64	26.253	Acetic acid methyl ester	-	>LOQ	>LOQ	>LOQ	>LOQ	>LOQ
65	26.424	Methoxyphenyl Oxime	-	>LOQ	>LOQ	>LOQ	>LOQ	>LOQ
66	27.770	1-Phenyl-1-butanone	3-heptanone	42.6 ± 4.4	109.2 ± 6.8	70.7 ± 2.4	96.1 ± 4.3	111.5 ± 14.6
67	28.022	(E,E)-2,4-Decadienal	Heptanal	90.4 ± 5.6	ND	81.6 ± 5.6	76.7 ± 6.5	ND
68	28.606	4-mehthy-2-phenyl1,3-dioxolane	-	ND	ND	>LOQ	>LOQ	ND
69	29.313	benzylalchocol *	1-heptanol	ND	ND	ND	190 ± 20	ND
70	30.090	Phenyl ethyl alcohol *	1-heptanol	52.5 ± 5.1	52.3 ± 9.8	101.4 ± 12.4	>LOQ	>LOQ
71	35.259	Nonadecane	Heptane	ND	ND	ND	38.5 ± 5.6	ND
72	37.050	Tricosane	Heptane	ND	ND	ND	10.5 ± 1.9	ND
73	38.942	Tetracosane	Heptane	ND	ND	ND	17.4 ± 1.7	ND
74	41.164	Hexacosane	Heptane	ND	ND	ND	± 3.6	ND

* Compounds of Almond Sample 1 that were detected, when performing extraction at a temperature of 37 °C in order to evaluate the volatiles that are responsible for the drink’s flavor.
